# C-Phycocyanin Alleviates Bladder Inflammation and Dysfunction in Cyclophosphamide-Induced Cystitis in a Mouse Model by Inhibiting COX-2 and EP4

**DOI:** 10.1155/2019/8424872

**Published:** 2019-07-29

**Authors:** Xing-qi Bao, Yi-chen Huang, Fang Chen

**Affiliations:** ^1^Department of Urology, Shanghai Children's Hospital, Shanghai Jiao Tong University, Shanghai, 200062, China; ^2^Shanghai 6th People's Hospital, Shanghai Jiao Tong University, Shanghai, 200233, China

## Abstract

**Objective:**

To explore the effect of C-phycocyanin (C-PC) on voiding behavior and histological changes in cyclophosphamide- (CYP-) induced cystitis in mice.

**Methods:**

Sixty female mice were included. The mice in the C-PC group received C-PC (25 mg/kg, twice, i.p.) and then CYP (200 mg/kg, i.p.) two hours later, while the mice in the CYP group only received the equivalent CYP. Saline was injected in the mice in the control group. A voided stain on paper (VSOP) test was conducted to analyze the micturition. The bladders were harvested for histological evaluation and measurements of inflammatory factors.

**Results:**

C-PC reduced the micturition frequency in the mice with CYP-induced cystitis. The bladder/body weight ratio and edema were remarkably higher in the CYP group compared to the C-PC group. C-PC suppressed the expressions of COX-2, PGE_2_, and EP4 (prostaglandin E receptor 4) according to the ELISA assay. Immunohistochemical staining also indicated that C-PC reduced the expressions of COX-2 in urothelium and EP4 in smooth muscles.

**Conclusions:**

C-PC relieved symptoms associated with CYP-induced cystitis in mice by inhibiting bladder inflammation through COX-2 and EP4 expression.

## 1. Introduction

Bladder pain syndrome/interstitial cystitis (BPS/IC) is defined by suprapubic chronic pain and/or discomfort associated with bladder storage without well-established pathogenesis such as urinary tract infection or neoplasm. Although there are many treatments for BPS/IC, their efficacies are barely satisfactory [1].

Prostaglandin E2 (PGE_2_) is a cytoprotective compound that is upregulated in tumors, bladder overactivity, and urinary tract infection [2]. It was revealed that the level of PGE_2_ was negatively correlated with the bladder capacity, and its overexpression may contribute to the process of hypersensitivity and inflammation by acting on its receptor to modulate the excitability of capsaicin-sensitive bladder afferents [3]. Cyclooxygenase-2(COX-2), which catalyzes the conversion of arachidonic acid to prostaglandins, especially PGE_2_ in the lower urinary tract, was shown to be involved in the pathogenesis of BPS/IC in a murine model [4]. Intravesical botulinum toxin A has proven to efficiently suppress bladder hyperactivity by preventing the elevation of PGE_2_ and COX-2 in cyclophosphamide- (CYP-) induced cystitis in rats [5]. However, adverse events, including urine retention and subsequent infections can occur.

C-phycocyanin (C-PC) is obtained from Spirulina platensis [6]. As a new COX-2 inhibitor, many studies have identified anti-inflammatory and neuroprotective activity of C-PC that result from directly inhibiting COX-2 expression and related inflammatory factors [7]. Dietary supplementation with Spirulina platensis also significantly downregulated the expression of COX-2 in a mouse model of salicylate-induced tinnitus[8]. Based on the known benefits of C-PC, we aimed to evaluate the potential of C-PC in treating CYP-induced cystitis in mouse model.

## 2. Materials and Methods

### 2.1. Animal Preparation

**Figure 1:**
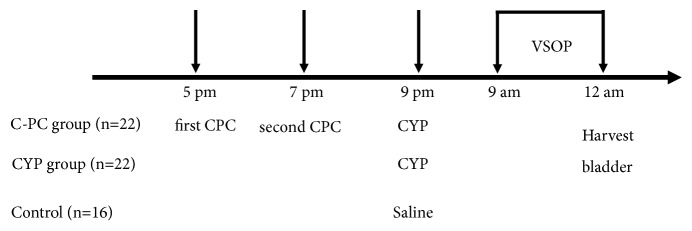
*Protocol for animal preparation*. VSOP was performed between 9 am and 12 am on the following day. The bladder was harvested following the micturition test.

All protocols were approved by Shanghai Jiao Tong University School of Medicine (IACUC: B-2018-002). Sixty C57BL/6 7- to 8-week-old female mice (18 to 24 g) were obtained from SIPPR-BK Laboratory Animals Co., Ltd. (Shanghai, China). The mice were randomly divided into three groups (control, CYP, and C-PC group). The mice in the C-PC group received C-PC twice (25 mg/kg with 2-hour interval, i.p., Sigma-Aldrich) and then CYP (200 mg/kg, i.p., Baxter) two hours later. The mice in the CYP group only received the equivalent CYP. Sterile saline was injected into the mice in the control group at the same time. The purity value of C-PC was 3.5, as determined by the A620/A280 absorbance ratio. The urinary frequency test was performed 12 h after the final treatment. The timing of the injections was kept consistent to minimize the chance for error and bias (Figure 1).

### 2.2. VSOP

The mice were kept in individual cages with filter paper lining the bottom (Solarbio, China) for 3 hours. During the test, the mice were fed with dry mouse chow, but water was withheld. The number of urinary spots was examined and recorded using an ultraviolet light camera (UVP BioSpectrum Imaging System, USA). ImageJ software (NIH, USA) was used to analyze the spots. Urinary volume is linearly associated with the area of the spot (R=0.9989). Sporadic noncircular small-diameter urine spots (particle<0.01475 cm^2^, corresponding to 0.5 *μ*L urine) were excluded.

### 2.3. Histology and Immunofluorescence (IF)

The bladders (control n=8, CYP n=11, C-PC n=11) were harvested through a lower midline abdominal incision from the bladder neck after the VSOP test. The body weight (g) and the emptied bladder wet weight (mg) were recorded. Bladder was divided along the vertical axis into two equal parts; one half was embedded into paraffin for HE staining, and the other half was prepared in a 30% sucrose solution overnight for dehydration.

Five serial paraffin-embedded bladder sections (3 *μ*m) were stained using the standard HE procedures. The assessment of bladder inflammation was based on four aspects (hemorrhage, urothelium lesion, edema, and leukocyte infiltration) as described by Cayan [9]. For the counting of mast cell, sections were deparaffinized, hydrated, and then incubated for 10 min in working Toluidine Blue solution (0.1%). The mean number of mast cells in 4 consecutive sections was recorded for the comparison of mastocytosis.

The other half of the bladder, which was stored in the sucrose solution, was embedded in OCT for immunofluorescence (IF). Bladder sections (8 *μ*m) cut with a cryostat were blocked with 10% goat serum for 60 min at room temperature. The colocalization of COX-2 and EP4 was detected as follows: blocked sections were incubated with a rabbit polyclonal anti-COX-2 antibody (1:100 dilution, Proteintech) and a mouse monoclonal anti-EP4 antibody (1:200 dilution, Santa Cruz) at 4°C overnight. To investigate the location of EP4 and TRPV1, sections were incubated with mouse monoclonal anti-EP4 (1:200, Santa Cruz) and rabbit polyclonal anti-TRPV1 (1:200 dilution, Proteintech) overnight at 4°C. After being washed, the slides were incubated with goat anti-mouse IgG conjugated with Cy3 and donkey anti-rabbit IgG conjugated with 488 (Boster, Wuhan, China) for 60 min at room temperature. Later, DAPI staining was applied for 6 min. Finally, each slide was covered with antifade mounting medium. The negative control was treated without the primary antibody.

### 2.4. Determination of the Levels of Inflammatory Factors Level in the Bladder

The intact bladder (control n=8, CYP n=11, C-PC n=11) was harvested and divided into two equal parts for Elisa and RT-PCR. Briefly, one part of bladder was minced and homogenized in 5% EDTA/NaCl buffer (pH 4.7), and the resulting homogenate was prepared for detection of the levels of TNF-*α*, IL-1*β*, IL-6, PGE_2_, COX-2, and EP4 through ABC-Elisa assay. The absorbance (OD) was determined within 30 minutes, using microplate reader set to 450 nm.

### 2.5. Neutrophil Myeloperoxidase (MPO) Activity Assay

The procedures were performed according to the manufacturer's recommendations (Nanjing Jiancheng Bioengineering Institute, China). The OD was measured at 460 nm and used in the following formula to calculate MPO. No samples fell below the detection limits of the assays:

(1) $$\begin{array}{c} MPO\left(\;U/g\;\right)=\frac{{OD}_{\left(simple\right)}-{OD}_{\left(control\right)}}{11.3\times simple\;\;weight\left(g\right)} \end{array}$$

### 2.6. Quantitative Real-Time PCR

Total RNA of half bladder was extracted using TRIzol reagent (Invitrogen Life Technologies, USA). One microgram of RNA was reversely transcribed with PrimeScript RT Master (Takara, Japan). Primers of* Cox-2* (F: CTCCACCGCCACCACTAC, R: GCAAGGATTTGCTGCCTGG) and* Ep4* (F: CCATTCCCGCAGTGATGTTCA, R: TGCGCGACTTGCACAATACTA) were used for amplification, and* Gapdh* (F: AGGTCGGTGTGAACGGATTTG, R: GGGGTCGTTGATGGCAACA) was chosen for housekeeping gene to normalize the expression levels using ∆∆CT method.

### 2.7. Statistical Analysis

All values were reported as the mean ± SEM except for specific indications. Differences between two groups were analyzed using a t-test, and the three groups were analyzed by one-way ANOVA as appropriate, or a nonparametric test (GraphPad Prism 6, USA). A p value < 0.05 was deemed to be significant statistically.

## 3. Results

### 3.1. C-PC Relieved the Bladder Inflammation Caused by CYP

**Figure 2:**
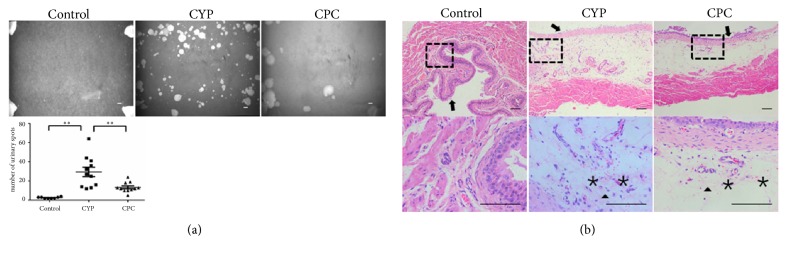
*C-PC's influence on CYP-induced cystitis*. (a) Representative photos of the VSOP. The number of urinary spots in the C-PC group was significantly decreased compared with CYP group. *∗∗* p<0.01. Scale bar 1 cm. (b) HE staining of the bladder. CYP induced severe bladder inflammation, and C-PC attenuated this effect. In normal bladder, the urothelium (arrow) was intact, and there was no edema within the lamina propria. Hemorrhage (asterisk) was not observed, and few leukocytes (triangle) infiltrated the blood vessels. Most of the epithelium layer was exfoliated in CYP group, and the lamina propria became thicker. Massive hemorrhage and leukocyte infiltration could be observed in the lamina propria and smooth muscles. In C-PC group the lamina propria was thinner and hemorrhage and leukocyte infiltration were more localized. Scale bar 100 *μ*m in all panels.

The number of voiding spots in the CYP group increased 11.3 folds compared with the control group (p=0.0002) (Figure 2(a)). The pretreatment with C-PC significantly reduced the spots number by 2 folds when compared with the CYP group (p=0.0051).

According to HE staining (Figure 2(b)), the bladder of the saline-treated mice (control group) presented normal histological features of noninflammation; neither edema nor hemorrhage was observed, and few leukocytes infiltrated the outsides of the vessels. After CYP treatment, severe cystitis appeared, including serious hemorrhage, wide edema, and leukocyte infiltration. In contrast, pretreatment with C-PC resulted in mild hemorrhage and edema and less severe mucosal lesions.

Bladder in the CYP-treated mice received the highest score in most aspects (Table 1). C-PC intervention significantly decreased edema (p=0.007) and leukocytes infiltration (p=0.0251) and showed a downward trend in hemorrhage. Moreover, the ratio of bladder-to-body also decreased significantly after C-PC treatment (p=0.0015). Only a few mastocytes were scattered throughout the bladder in each group, and C-PC pretreatment reduced the number of mast cells significantly compared with CYP group (from 8.909 ± 1.268 to 5.273 ± 0.4491, p=0.0137). Treatment with CYP increased the number of mast cells 3.56 folds (p<0.0001) compared to the control; however, the C-PC clearly exhibited lower numbers compared with the CYP group (p=0.0137).

**Figure 3:**
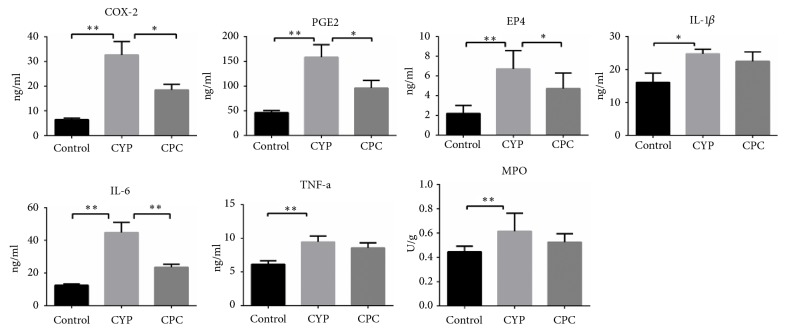
*The effect of C-PC on the expression of bladder inflammatory factors by ELISA*. Pretreatment with C-PC suppressed the expression of PGE_2_, IL-6, and MPO compared with CYP treatment. *∗* p <0.05, *∗∗* p<0.01.

C-PC inhibited the expression of inflammatory factors in bladder (Figure 3). The level of all factors increased significantly in whole bladder after CYP treatment compared to the saline group, including PGE_2_ (3.42 folds), IL-1*β* (1.39 folds), IL-6 (4.13 folds), and TNF-*α* (3.5 folds). Pretreatment with C-PC reduced the expressions of COX-2 (p=0.0263), EP4 (p=0.0143), PGE2 (p=0.0474), and IL-6 (p=0.0038) significantly compared with the CYP group. But C-PC did not significantly reduce MPO activity (p=0.2475).

### 3.2. C-PC Suppressed the Expressions of COX-2 and EP4 in Bladder

**Figure 4:**
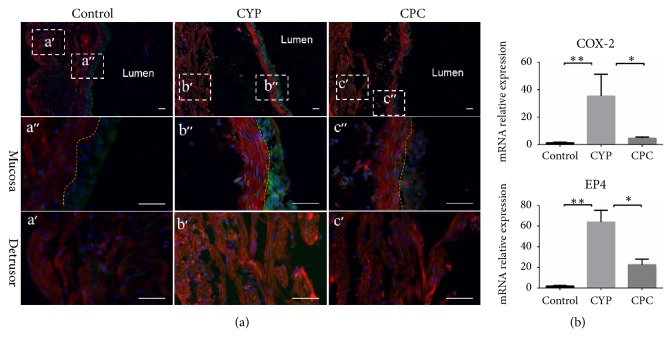
*The expressions of COX-2 and EP4 in bladder*. (a) The locations of COX-2 (green) and EP4 (red) in the bladder. The control group exhibited weak COX-2 -IR and EP4 -IR signals in the mucosa and detrusor, respectively. The CYP group exhibited stronger COX-2 -IR and EP4 -IR in the same location, and pretreatment with C-PC also resulted in the visible intensity of COX-2 -IR and EP4 -IR. Yellow dotted lines delineated the boundary of urothelium. Scale bar 25 *μ*m. (b) mRNA relative expression. Transcription levels of* Cox-2* and* Ep4* increased significantly after CYP treatment, and C-PC reduced the expressions of those genes. *∗* p <0.05, *∗∗* p<0.01.

The expressions of COX-2 and EP4 were evaluated to investigate the potential mechanism. In the bladder of the saline-treated mice, there were only weak COX-2-IR (green stain) and EP4-IR (red stain) signals in the urothelium and suburothelium & detrusor, respectively (Figure 4(a)). However, the COX-2 positive cells were observed primarily in urothelium and scarcely in suburothelium in the CYP group and the C-PC group after the CYP injection. Moreover, EP4-IR was obviously stronger in the SMC layer and the suburothelium after the CYP injection. Pretreatment with C-PC reduced the intensity of COX-2- and EP4 -IR in corresponding localizations (Figure 3(a)). The expression of* Cox-2* and* Ep4* also decreased as transcript level (Figure 4(b)). Overall, the expression levels and locations of COX-2 and EP4 were different in the mouse bladder, and C-PC suppressed the expressions of both.

### 3.3. The Altered Expression of TRPV1 in Bladder

**Figure 5:**
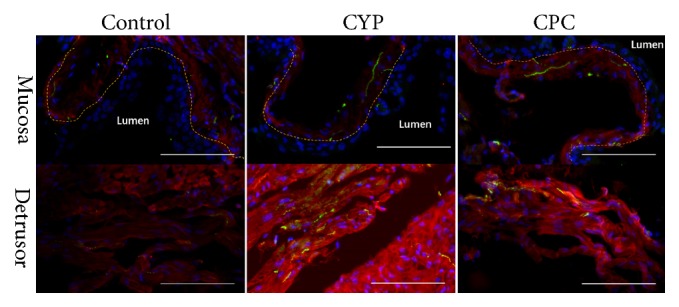
*The expressions of TRPV1 and EP4 in bladder*. EP4 (red) and TRPV1 (green) innervation in lower urinary bladder. EP4-IR and TRPV1-IR signals were weak in the control group. EP4-IR was primarily localized to the detrusor muscle, and TRPV1-IR primarily passed through the suburothelium and smooth muscle cells following CYP treatment. Yellow dotted lines delineated the boundary of the urothelium. Scale bar 50 *μ*m in the panels.

TRPV1 in the lower urinary tract is a classical receptor that induces excitability of the C-afferent. The TRPV1-IR (green stain) nerve fiber passed through the suburothelium and the EP4-stained (red stain) bladder detrusor muscle, but not the urothelium (Figure 5). TRPV1-IR was more obvious after CYP treatment in contrast to control; however, the influence of C-PC on TRPV1-IR intensity in the C-PC group was also remarkable, which was consistent with the analysis of real-time PCR.

## 4. Discussion

Patients suffering from BPS/IC are usually associated with a decreased quality of life due to the lack of effective treatments. In our study, we aimed to determine the effects of C-PC on voluntary urination and bladder inflammation in consideration of the proven effects of C-PC. Our research first demonstrated that C-PC benefits the mouse model with BPS/IC by mitigating voiding symptoms and resolving bladder inflammation.

Numerous studies have shown that C-PC has promising prospect due to its multiple-biological functions [7]. Ricardo et al. [10] found that the oral administration of phycocyanin reduced inflammatory cell infiltration and microvilli loss in acetic acid-induced colitis in rats. This finding demonstrated that C-PC has anti-inflammation activity in mucosal disease. Saini et al. [11] revealed the role of C-PC in inhibiting COX-2 by comparing C-PC with piroxicam, which is a traditional NSAID. Both of those compounds reduced the number and size of tumors in dimethylhydrazine dihydrochloride-induced rat colon cancer. In the present study, we revealed that C-PC administration also exerted an anti-inflammatory effect in CYP-induced cystitis; C-PC mitigated the inflammatory appearance and scores of the bladder, including edema and leukocytes infiltration. Moreover, C-PC significantly reduced the levels of PGE_2_ and IL-6 in the bladder. We further investigated the relations of COX-2 and EP4, and we found that both genes are expressed at low levels in normal bladder. Unlike COX-2, which is primarily localized to the bladder mucosa, EP4 is upregulated primarily in the smooth muscle layer and the suburothelium. Our findings may suggest that the overexpression of EP4 in the rodent bladder mediates the action of PGE_2_ produced by urothelium.

VSOP was performed to determine the frequency of micturition. Urinating on paper is a noninvasive method compared with urodynamic testing [12]. The results of VSOP suggested that C-PC has antihyperalgesia activity, which is in agreement with Chou's result that C-PC played a preventative role in inflammatory nociception in rats with carrageenan-evoked thermal hyperalgesia [13]. The author demonstrated that C-PC downregulated the expression of NO and PGE_2_ through inhibiting iNOS and COX-2 induction in a dose-dependent manner. PGE_2_ and the subsequent EP-receptor subtypes have been investigated for their contributions to allodynia and hyperalgesia by stimulations of various PGE_2_ analogues [14]. Hu et al. [3] found that COX-2 and its metabolites also play an important role in bladder overactivity in CYP-induced cystitis. Further research uncovered that the CYP-induced upregulation of EP4 involved the detrusor and led to dysfunction of the micturition reflex [15]. The intravesical administration of botulinum toxin A suppressed bladder hyperactivity and inflammation in CYP-stimulated cystitis by downregulating the expressions of COX-2 and EP4, which are both upregulated in bladder and spinal cord after CYP treatment [5]. Further research revealed that a selective EP4 antagonist increased the intercontraction interval in PEG_2_-induced hyperactivity and had no significant effects on the control group [16].

It is interesting to find that the TRPV1-IR nerve fiber passed through both the EP4-positive SMC and the suburothelium. This may suggest that the overexpression of EP4 in the rodent bladder mediates the action of PGE_2_ on TRPV1, which is expressed in C-afferent, to induce bladder overactivity. Although the mechanism of action of EP4 in bladder was not clear, TRPV1 may be involved in the pathological process of bladder dysfunction. Moriyama et al. [17] reported that PGE_2_ could act on both EP1 and EP4 to potentiate TRPV1 activity via PKC- and PKA-dependent pathways, respectively, and both EP1 and EP4 contribute to the development of persistent hyperalgesia and inflammation of the dorsal root ganglion. Ma et al. [18] study has revealed that an EP4 agonist induced TRPV1 externalization and was suppressed by inhibitors of the cAMP/PKA and ERK/MAPK signal pathways. It is strongly implied that the PEG_2_-EP4 -TRPV1 axis mediates bladder overactivity in CYP-induced cystitis in the murine bladder via the abovementioned signaling pathway. More works are needed to explore this pathway in the future.

## 5. Conclusions

In conclusion, developing treatment for BPS/IC is a tough nut to crack. This work demonstrated that C-PC can alleviate bladder inflammation and improve the dysfunction by inhibiting COX-2 expression. C-PC may be a potential option for daily management of BPS/IC.

## Figures and Tables

**Table 1:** The inflammatory scores and weight ratio in the three groups.

	control (n=8)	CYP (n=11)	CPC (n=11)	p(control vs CYP)	p(CYP vs CPC)	p(three groups)
hemorrhage	27.52 ± 8.467	81.01 ± 5.606	66.46 ± 5.087	< 0.0001	0.069	< 0.0001
urothelium damage	26.48 ± 8.253	86.85 ± 9.055	92.80 ± 6.054	< 0.0001	0.5908	< 0.0001
edema	14.58 ± 6.379	72.47 ± 6.378	46.54 ± 5.321	0.0002	0.007	0.0002
leukocyte infiltration	18.61 ± 4.227	40.70 ± 3.303	33.66 ± 5.654	0.0003	0.0251	0.0018
mastocytes	2.500 ± 0.1890	8.909 ± 1.268	5.273 ± 0.4491	< 0.0001	0.0137	< 0.0001
Bladder(mg)/body(g)	0.9456 ± 0.07006	2.183 ± 0.1994	1.568 ± 0.08215	< 0.0001	0.0015	< 0.0001

all values were presented in mean ± SEM.						

All indicators were of significance among the three groups.

## Data Availability

(1) The data of VSOP used to support the findings of this study are included within the article. (2) The histological results used to support the findings of this study are included within the article. (3) The immunofluorescence results used to support the findings of this study are included within the article. (4) The data of Elisa used to support the findings of this study are included within the article. (5) The data of qRT-PCR used to support the findings of this study are included within the article. (6) The methods of evaluation of bladder inflammation used to support the findings of this study have been deposited in the Cayan repository (PMID: 12131370).
